# About the International Conference on Emerging Infectious Diseases

**DOI:** 10.3201/eid0403.980301

**Published:** 1998

**Authors:** Stephen A. Morse

**Affiliations:** Centers for Disease Control and Prevention, Atlanta, Georgia, USA


### About the International Conference on Emerging Infectious Diseases

**Stephen A. Morse**
Centers for Disease Control and Prevention, Atlanta, Georgia, USA

More than 2,500 researchers, clinicians, laboratorians, veterinarians, and other public health professionals from all 50 states and more than 70 countries convened in Atlanta on March 8-11, 1998, for the International Conference on Emerging Infectious Diseases. The conference, organized by the Centers for Disease Control and Prevention (CDC), the Council of State and Territorial Epidemiologists, the American Society for Microbiology, and the National Foundation for CDC along with 62 other cosponsors,[1] provided a forum for the exchange of ideas and possible solutions to the problems of new and reemerging infectious diseases, including potential threats presented by bioterrorism. Several agencies and organizations sponsored satellite partnership meetings on March 8 and March 12.

More than 85 sessions (12 plenary sessions, 17 invited panels, 35 poster sessions, and late-breaking abstracts) were presented on surveillance, epidemiology, prevention, and control of emerging infectious diseases, as well as emergency preparedness and response and reemerging or drug-resistant infectious diseases. Topics included foodborne diseases, infectious diseases transmitted by animals and insects, nosocomial infections, infections in immunocompromised patients and persons outside the health-care system, infectious causes of chronic disease, blood safety, host genetics, vaccines, global climate change, and immigration and travel.

In delivering the keynote address, Nobel laureate Joshua Lederberg reviewed the scientific basis for the emergence of infectious diseases. U.S. Health and Human Services Secretary Donna Shalala and Assistant Secretary for Health and Surgeon General David Satcher, along with representatives from the World Health Organization, the Pan American Health Organization, and the U.S. Agency for International Development, and representatives from academia and industry addressed the national and international ramifications of emerging infections. In closing the conference, James Hughes, director, National Center for Infectious Diseases, CDC, stressed the importance of building bridges and forging new partnerships to prevent and control the emergence of infections into the next millennium.

In publishing the conference presentations and discussions in this journal, the organizers hope to capture the energy expressed by all participants, further disseminate new information on emerging infections, and stimulate more research and other initiatives against this important public health threat.

---

[1]Alliance for the Prudent Use of Antibiotics, American Academy of Pediatrics, American Association of Blood Banks, American Association of Health Plans, American Cancer Society, American College of Preventive Medicine, American Hospital Association, American Medical Association, American Mosquito Control Association, American Public Health Association, American Sexually Transmitted Diseases Association, American Society of Clinical Pathologists, American Society of Tropical Medicine and Hygiene, American Veterinary Medical Association, Association of American Veterinary Medical Colleges, Association of Schools of Public Health, Association of State and Territorial Directors of Health Promotion and Public Health Education, Association of State and Territorial Health Officials, Association of State and Territorial Public Health Laboratory Directors, Association of Teachers of Preventive Medicine, Burroughs Wellcome Fund, Emory University School of Medicine, Fogarty International Center, Food and Drug Administration, Indian Health Service, Infectious Diseases Society of America, International Life Sciences Institute, International Society for Infectious Diseases, International Society of Travel Medicine, International Union for Health Promotion and Education, International Union of Microbiological Societies, Minority Health Professions Foundation, Morehouse School of Medicine, National Aeronautics & Space Administration, National Association of City and County Health Officials, National Association of State Public Health Veterinarians, National Council for International Health, National Foundation for Infectious Diseases, National Hispanic Medical Association, National Institute of Allergy and Infectious Diseases, National Medical Association, National Oceanographic & Atmospheric Administration, Office of Science and Technology Policy, Pan American Health Organization, Rollins School of Public Health of Emory University, Society for Healthcare Epidemiology of America, Society for Occupational and Environmental Health, Society for Public Health Education, The Carter Center, The Henry J. Kaiser Family Foundation, The HMO Group, The Robert Wood Johnson Foundation, The Rockefeller Foundation, The World Bank, U.S. Agency for International Development, U.S. Department of Agriculture, U.S. Department of Defense, U.S. Department of State, U.S. Department of Justice (INS), U.S. Department of Veterans Affairs, U.S. Environmental Protection Agency, World Health Organization.

More than 2,500 researchers, clinicians, laboratorians, veterinarians, and other public health professionals from all 50 states and more than 70 countries convened in Atlanta on March 8-11, 1998, for the International Conference on Emerging Infectious Diseases. The conference, organized by the Centers for Disease Control and Prevention (CDC), the Council of State and Territorial Epidemiologists, the American Society for Microbiology, and the National Foundation for CDC along with 62 other cosponsors,^1^ provided a forum for the exchange of ideas and possible solutions to the problems of new and reemerging infectious diseases, including potential threats presented by bioterrorism. Several agencies and organizations sponsored satellite partnership meetings on March 8 and March 12.

More than 85 sessions (12 plenary sessions, 17 invited panels, 35 poster sessions, and late-breaking abstracts) were presented on surveillance, epidemiology, prevention, and control of emerging infectious diseases, as well as emergency preparedness and response and reemerging or drug-resistant infectious diseases. Topics included foodborne diseases, infectious diseases transmitted by animals and insects, nosocomial infections, infections in immunocompromised patients and persons outside the health-care system, infectious causes of chronic disease, blood safety, host genetics, vaccines, global climate change, and immigration and travel.

In delivering the keynote address, Nobel laureate Joshua Lederberg reviewed the scientific basis for the emergence of infectious diseases. U.S. Health and Human Services Secretary Donna Shalala and Assistant Secretary for Health and Surgeon General David Satcher, along with representatives from the World Health Organization, the Pan American Health Organization, and the U.S. Agency for International Development, and representatives from academia and industry addressed the national and international ramifications of emerging infections. In closing the conference, James Hughes, director, National Center for Infectious Diseases, CDC, stressed the importance of building bridges and forging new partnerships to prevent and control the emergence of infections into the next millennium.

In publishing the conference presentations and discussions in this journal, the organizers hope to capture the energy expressed by all participants, further disseminate new information on emerging infections, and stimulate more research and other initiatives against this important public health threat.

## Footnotes

1 Alliance for the Prudent Use of Antibiotics, American Academy of Pediatrics, American Association of Blood Banks, American Association of Health Plans, American Cancer Society, American College of Preventive Medicine, American Hospital Association, American Medical Association, American Mosquito Control Association, American Public Health Association, American Sexually Transmitted Diseases Association, American Society of Clinical Pathologists, American Society of Tropical Medicine and Hygiene, American Veterinary Medical Association, Association of American Veterinary Medical Colleges, Association of Schools of Public Health, Association of State and Territorial Directors of Health Promotion and Public Health Education, Association of State and Territorial Health Officials, Association of State and Territorial Public Health Laboratory Directors, Association of Teachers of Preventive Medicine, Burroughs Wellcome Fund, Emory University School of Medicine, Fogarty International Center, Food and Drug Administration, Indian Health Service, Infectious Diseases Society of America, International Life Sciences Institute, International Society for Infectious Diseases, International Society of Travel Medicine, International Union for Health Promotion and Education, International Union of Microbiological Societies, Minority Health Professions Foundation, Morehouse School of Medicine, National Aeronautics & Space Administration, National Association of City and County Health Officials, National Association of State Public Health Veterinarians, National Council for International Health, National Foundation for Infectious Diseases, National Hispanic Medical Association, National Institute of Allergy and Infectious Diseases, National Medical Association, National Oceanographic & Atmospheric Administration, Office of Science and Technology Policy, Pan American Health Organization, Rollins School of Public Health of Emory University, Society for Healthcare Epidemiology of America, Society for Occupational and Environmental Health, Society for Public Health Education, The Carter Center, The Henry J. Kaiser Family Foundation, The HMO Group, The Robert Wood Johnson Foundation, The Rockefeller Foundation, The World Bank, U.S. Agency for International Development, U.S. Department of Agriculture, U.S. Department of Defense, U.S. Department of State, U.S. Department of Justice (INS), U.S. Department of Veterans Affairs, U.S. Environmental Protection Agency, World Health Organization.

